# Risk is dead. Long live risk

**DOI:** 10.1192/bjb.2025.10142

**Published:** 2025-10

**Authors:** Jacqueline Huber, Christian Greiner, Paco Prada, Matthew Large

**Affiliations:** MMed FRANZCP Emergency Psychiatrist Psychiatry, St Vincent’s Hospital, Sydney, Australia; Deputy Head Physician, Liaison Psychiatrry and Crisis Intervention Service Department of Psychiatry, Geneva University Hospital, Geneva, Switzerland; Head of Service Consultation Liaison Psychiatry and Crisis Intervention Service, Geneva University Hospital, Geneva, Switzerland; FRANZCP, DMedSci Conjoint Professor Discipline of Psychiatry and Mental Health, University of NSW, Sydney, Australia

We write to draw attention to the recent NHS England publication *Staying Safe from Suicide: Best Practice Guidance for Safety Assessment, Formulation and Management*. This important document takes a principled and much-needed stance in rejecting the continued use of stratified suicide risk assessment tools, highlighting their poor predictive validity and potential to undermine care. The document marks a significant cultural change, encouraging practitioners to relinquish false assurances in favour of more meaningful and relational modes of care.

However, we are concerned that the guidance’s language at times risks undermining its central message. Specifically, although risk stratification is clearly (and appropriately) rejected, the guidance continues to require clinicians to undertake a ‘risk formulation’ as part of every psychosocial assessment. This phrase is not defined in the document, yet its juxtaposition with language of ‘safety assessment’ and identification of ‘future risk factors’ and ‘protective factors’ suggests that the intention may still be to assess and manage individual suicide risk, albeit using different tools and methods. This creates tension between the document’s recognition that suicide risk cannot be reliably predicted and its continued emphasis on formulations framed in terms of ‘risk’. In our view, this may inadvertently reinforce outdated assumptions that clinicians can and should manage suicide risk directly.

We suggest that a more helpful framing would be to move away from ‘risk formulation’ altogether and instead adopt language centred on clinical need, distress and collaborative development of a shared understanding. Formulation remains an essential clinical task, but not because it predicts suicide. Rather, it offers an opportunity to understand suffering, identify sources of support, and co-produce plans to reduce distress and improve care.

As one lived experience partner eloquently states in the document: ‘We are people, not just a risk that must be managed’. The guidance aspires to embody this ethos, yet we worry that legacy terminology – particularly the reference to ‘risk formulation’ – risks reintroducing the very constructs it seeks to dismantle.

We encourage NHS England and other organisations adopting this guidance to ensure that clinical language reflects the paradigm shift they advocate: away from prediction and management of risk and towards compassionate, relational, person-centred care.

